# Supplementary material for: Beyond ratios - flexible and resilient nurse staffing options to deliver cost-effective hospital care and address staff shortages: A simulation and economic modelling study
Source: Int J Nurs Stud. 2021 May;117:103901. doi: 10.1016/j.ijnurstu.2021.103901 (PMC8220646; doi:10.1016/j.ijnurstu.2021.103901)
Supplement: Supplementary file 1 [file mmc1.docx]

**Beyond ratios - flexible and resilient nurse staffing options to deliver cost-effective hospital care and address staff shortages: a simulation and economic modelling study****:** Supplementary material for online publication.

# Appendix 1 Simulation model technical details (page 2)

# Appendix 2 Additional Tables (page 6)

# Appendix 1 Simulation model technical details

*Reproduced with permission from: Appendix 1* Griffiths, P., Saville, C., Ball, J.E., Chable, R., Dimech, A., Jones, J., Jeffrey, Y., Pattison, N., Saucedo, A.R., Sinden, N., Monks, T., 2020. The Safer Nursing Care Tool as a guide to nurse staffing requirements on hospital wards: observational and modelling study. Health Serv Deliv Res 8, 16. (Changes to original: abbreviations spelled out and cross-references amended.)

### Implementation and experimentation

The simulation was developed on a Windows 10 Enterprise operating system version 1709, build number 16299.726 in Anylogic 8 researcher edition software version 8.3.2, build number 8.3.2.201807061745 x64. This used Java 2 Standard Edition 8.0 and Fluid library version 2.0.0.

We used the default random number generator in Anylogic: an instance of the Java class Random, which is a Linear Congruential Generator (LCG).

Time is modelled as fixed time steps. There are no concurrent events that are interdependent.

There is no warm-up period or model initialisation since each day/shift is independent of the next. The model is stochastic. The run length is one year, and the time units are six-hour shifts (the model also works for days). The number of replications is 10.

Running one experiment (1 year, 10 runs) takes between 10 and 20 minutes for the different hospital Trusts on a Dell Latitude laptop with Intel® Core™ i5-7300U processor with 2.60GHz CPU speed, 8 GB RAM and 32 GB ROM.

### Computer Model Sharing Statement

For a video demonstration see https://eprints.soton.ac.uk/430632/. The simulation model is available from the corresponding author on reasonable request. Anylogic simulation software can be downloaded from https://www.anylogic.com/downloads/.

### Simulation model logic

An overview of the simulation logic is shown in Figure 2. Each stage is described in more detail below. The simulation was designed to work for any number of wards. The wards are ‘agents’ which interact with each other (within directorates) by lending/borrowing staff in an attempt to cover shortfalls of staffing in one ward with surplus in another. Outputs are updated at the end of each time step (day/shift) and aggregated at the end of each year.

Figure 2 Simulation model overview diagram

#### Before running the simulation

Before running the simulation for a particular hospital Trust, information about its wards is imported from Excel files to fill database template tables within the simulation. The three files contain occupancy data, acuity/dependency data and other ward information. The occupancy distributions consist of the number of patients, labelled with the ward identification numbers (a contiguous number for internal purposes and the number used for external reporting), day of the week and 6-hour-shift. The acuity/dependency distributions consist of the proportions of patients in each SNCT acuity/dependency level and the proportion of patients that require specialing, labelled with the ward identification numbers and observation number (used when sampling a row). The other ward information consists of the baseline staffing levels for each scenario (in WTE), the skill mix (in the morning, afternoon, evening, night, and over a 24-hour period), the distribution of staff over 24 hours (proportion of staff deployed in the morning/afternoon/evening/night), the directorate number, whether the ward is an acute admissions unit and some purely descriptive information (directorate, medical or surgical).

The probabilities of requests for temporary staff being fulfilled is imported from an Excel file into another database template table. The template allows probabilities to differ between bank and agency, up to three staff types, weekend and weekday, and time period (morning, afternoon, evening, night, and over a 24-hour period).

Next, further parameter values that apply globally to the whole hospital Trust (the system) are set. These parameters relate to:

(1) nursing staff requirements - acuity/dependency multipliers, specials multiplier, standard deviation of multipliers, minimum number of registered nurses needed (constraint), demand for registered nurses with a particular skill

(2) permanent staff - the baseline staffing level, the data sample used to calculate the staffing level, the staff types, absence chance for each staff type, absence length, proportion of registered nurses with a particular skill

(3) redeployed staff - redeployment rules, priority sequences for providing and receiving redeployed staff, efficiency of redeployed staff, redeployed staff shift length

(4) temporary staff - rules for requesting temporary staff, efficiency of bank and agency staff, temporary staff shift length

(5) display settings - the understaffing criterion to plot in charts, e.g. 15% or more under requirement

(6) general settings - step length, round down bound used when converting required hours to requested hours.

#### At start of run

At the start of a run, variables tracking time (the step number and the shift number) are reset to zero. The wards, staff types and sharing groups are counted. Occupancy distributions are created from the occupancy data table. Data are placed in arrays, which are convenient structures for working with multi-dimensional data.

Then, the establishment (number of staff employed in WTE) is converted to the number of planned deployed hours per time step (6-hour-shift or day), including applying the skill mix, rounding to whole people and dealing with minimum constraints, as follows. Note that the establishment does not need to be a whole number since staff may work part-time, but the planned number of staff to deploy each six-hour shift should be a whole number. As in our other analyses we use equation 3 for converting the planned staffing in WTE to the planned total care hours per day (see Equation 3, Appendix 1, main report (Griffiths et al., 2020b).

The planned skill mix (proportion of staff that are registered nurses) and distribution of staff over the day in each ward is set as the average observed for that ward. There is a constraint that there must be at least one registered nurse present on each ward, so if the registered nurse hours is under 6, this is rounded up to 6. Otherwise, the registered nurse hours are rounded up or down to the nearest six hours. The remaining planned hours are assigned to nursing support workers, and again rounded up or down to the nearest 6 hours.

For example, suppose the planned nursing hours for a morning shift on a particular ward are 18, and the skill mix is 50%. This is equivalent to 9 hours of registered nurse time, which is rounded up to 12 hours. There are 6 hours left to cover which are assigned to nursing support workers.

The planned deployed hours per day (sum over the four shifts) are converted back into WTE to enable calculation of the cost of employing this number of permanent staff.

#### Before time-step

Before each time-step, i.e. before the simulation switches to the next period (six-hour shift or day), the variables for this period are updated. These variables are the time step, the shift (1 to 4), the day type (weekday, Saturday of Sunday/bank holiday) and the planned staffing level for this shift. The deployment array (numbers of staff from each source and of each staff type deployed on each ward in what capacity) is reset at zero ready to be filled in the next stages.

#### Before time-step, in each ward

Next, the required staffing for this period is calculated for each ward in turn. For this, firstly the number of patients on the ward is sampled from the occupancy distribution for that ward, day of week and shift (morning, afternoon, evening or night).

Secondly, the acuity/dependency profile is sampled from the acuity/dependency data. This is done by selecting a random observation for that ward (we assumed there were no day of week or time of day patterns). For each patient, the probability of being in each acuity/dependency category and the probability of requiring specialing are the corresponding observed proportions. The required staffing per patient (in WTE) is sampled based on the patient’s acuity/dependency category and specialing requirements. This is converted into the required staffing level for this period using the skill mix, distribution of staff over the day and minimum constraints (as for the planned staffing levels), but is not rounded.

#### On time-step, in each ward

On the time-step, i.e. immediately when the period begins, the number of hours of staffing provided by permanent staff in this period is calculated for each ward. For this, the number of planned staff who are not unexpectedly absent (i.e. at short notice) is calculated. The chance of being unexpectedly absent can differ between staff types in the model. All these staff are allocated to their home ward to start with. The (absolute) shortfall for each staff type is calculated as required minus allocated hours. Where applicable, the simulation checks which of the registered nurses working are IV-trained (sampled probabilistically).

Similarly the spare hours (hours that could be redeployed to another ward) for each staff type is calculated. This is the allocated minus the required hours, rounded down to the nearest multiple of ‘redeployed hours chunk’, since staff can only be redeployed for fixed time periods.

#### On time-step

Next, staff are redeployed within sharing groups (directorates) to attempt to cover shortfalls for each staff type, as shown in Figure 3. The shortfall is rounded up or down (depending on the round down bound) to the nearest multiple of ‘redeployment chunks’. Requests for extra staff are triggered if the rounded shortfall for that staff type is more than zero, and if either the total shortfall or the staff type shortfall are more than the trigger (6 hours). In order to decide the priority of redeploying extra staff to wards, lists of wards are sorted using the bubble sort algorithm(Knuth, 1981) , which works by comparing the shortfall as a proportion of the requirement (or spare hours as a proportion of requirement) for adjacent wards in the list and swapping them if they are in the wrong order.

Repeat until either there are no more wards requesting extra staff of this type, or there are no more wards with spare NH (for this staff type)

Figure 3 Summary process flow of staff redeployment

Following this, for the wards that are still requesting extra staff, for each staff type, first bank and then agency staff are requested, as shown in Fig 3 above. The hours requested are the shortfall rounded up or down (depending on the round down bound) to the nearest multiple of ‘external work time’. The probability of a request for temporary staff being fulfilled depends on the staff source, staff type, whether it is a weekday or weekend and the time period.

# Appendix 2 Additional Tables

Table 5 Observed percentage of same-day and previous-day temporary staff requests filled

| Requests fulfilled by bank staff | Registered nurses | Nursing assistants |
| --- | --- | --- |
| Weekday |  |  |
| Morning | 28% | 28% |
| Afternoon | 10% | 9% |
| Evening/night | 19% | 37% |
| Weekend |  |  |
| Morning | 32% | 31% |
| Afternoon | 13% | 17% |
| Evening/night | 19% | 32% |
| Requests fulfilled by agency staff |  |  |
| Weekday |  |  |
| Morning | 15% | 36% |
| Afternoon | 9% | 5% |
| Evening/night | 45% | 44% |
| Weekend |  |  |
| Morning | 19% | 43% |
| Afternoon | 14% | 14% |
| Evening/night | 37% | 39% |

Table 6 Registered nurse and nursing support worker costs (£) by Agenda for Change band (substantive and temporary staff)

| Agenda for change bands | Substantive staff | | | Bank staff | | | Agency staff | | |
| --- | --- | --- | --- | --- | --- | --- | --- | --- | --- |
|  | Salary a | Employer on cost | Total cost per hour b | Salary | Employer on costc | Total cost per hour b | Salary d (agency cap maximum) | Employer on cost | Total cost per hour e |
| 1 |  |  |  |  |  |  | 15,516 | 8,534 | 12.29 |
| 2 | 16,536 | 3,548 | 12.63 | 16,536 | 2,359 | 11.88 | 17,978 | 9,888 | 14.24 |
| 3 | 18,333 | 4,054 | 14.08 | 18,333 | 2,736 | 13.25 | 19,655 | 10,810 | 15.56 |
| 4 | 20,279 | 4,602 | 15.65 | 20,279 | 3,144 | 14.73 | 22,458 | 12,352 | 17.79 |
| 5 | 26,038 | 6,225 | 20.48 | 26,038 | 4,353 | 19.3 | 28,462 | 15,654 | 22.55 |
| 6 | 32,342 | 8,002 | 25.62 | 32,342 | 5,676 | 24.14 | 35,225 | 19,374 | 27.9 |
| 7 | 38,801 | 9,822 | 30.87 | 38,801 | 7,032 | 29.1 | 41,373 | 22,755 | 32.77 |
| 8a | 45,544 | 11,722 | 36.36 | 45,544 | 8,447 | 34.28 | 48,034 | 26,419 | 38.05 |
| 8b | 54,307 | 14,191 | 43.49 | 54,307 | 10,287 | 41.01 | 57,640 | 31,702 | 45.66 |
| 8c | 63,703 | 16,839 | 51.14 | 63,703 | 12,259 | 48.23 | 68,484 | 37,666 | 54.25 |
| 8d | 75,171 | 20,071 | 60.47 | 75,171 | 14,666 | 57.04 | 82,434 | 45,339 | 65.3 |
| 9 | 88,526 | 23,834 | 71.34 | 88,526 | 17,469 | 67.3 | 99,437 | 54,690 | 78.77 |
| A mean salary in 2017 as reported in unit costs of health and social care(Curtis and Burns, 2017)  B cost per hour based on annual hours in unit costs of health and social care(Curtis and Burns, 2017): 1,590 (42.4 weeks * 37.5) for band 2-4 and 1,575 (42 weeks * 37.5) for band 5 and above  C includes employer national insurance contribution and assumes 50% bank staff include employer superannuation payment  D salary is upper spine point of range for each band – method adopted in agency cap (NHS Improvement, 2018a)  E salary per hour based on annual hour calculation for agency cap (NHS Improvement, 2018a) – 52.18 weeks (365.25/7) * 37.5 = 1,956.75. | | | | | | | | | |

Table 7 Coefficients and baseline parameters used in cost-effectiveness models

| Parameter | Coefficient | | | Value^a^ | | |
| --- | --- | --- | --- | --- | --- | --- |
| Mortality^b^  effect [Upper, lower 95% confidence interval] | |  |  | | LCL | UCL |
| Day of Low registered nurse staffing | HR^c^ | | 1.03 | | [1.01, | 1.06] |
| Day of Low nursing support worker staffing | HR | | 1.04 | | [1.02, | 1.07] |
| Temporary staffing models^d^ |  | |  | |  |  |
| Day of Low registered nurse staffing | HR | | 1.03 | |  |  |
| Day of Low nursing support worker staffing | HR | | 1.05 | |  |  |
| Day of High temporary registered nurse staffing | HR | | 1.12 | |  |  |
| Day of High temporary nursing support worker staffing | HR | | 1.05 | |  |  |
| Baseline Mortality Rate^e^ |  | |  | |  |  |
| Hospital Trust A | % | | 3.21 | |  |  |
| Hospital Trust B | % | | 3.87 | |  |  |
| Hospital Trust D | % | | 3.15 | |  |  |
| Length of stay effect^b^ |  | |  | |  |  |
| Average registered nurse staffing relative to mean | β (raw) | | -0.23 | |  |  |
| Average nursing support worker staffing relative to mean | Β (raw) | | 0.076 | |  |  |
| Mean Length of Stay (in 5 days)^f^ |  | |  | |  |  |
| Hospital Trust A | days | | 3.65 | | (2.89) |  |
| Hospital Trust B | days | | 6.33 | | (3.74) |  |
| Hospital Trust D | days | | 5.05 | | (3.15) |  |
| Cost of excess bed day^g^ |  | |  | |  |  |
|  | £ | | 337 | |  |  |
| ^a^ Values reported here are rounded, however for the modelling reported here, we used precise values as originally calculated.  ^b^ Derived from Griffiths et al. (2018)  ^c^ HR Hazard Ratio  ^d^ As the temporary staffing models also included the effect of low staffing in the original analysis we used the low staffing coefficients from the model that generated the temporary staffing coefficients, hence the slight differences.  ^e^NHS Digital (2018b)  ^f^NHS Digital (2018a)  ^g^NHS Improvement (2018b) | | | | | | |

Table 8 Changes in Costs, effects and cost-effectiveness for resilient and flexible establishments relative to ‘standard’ establishments for varying levels of temporary staff availability without the use of float staff

|  | Staff cost | |  | Hospital stay | |  | Death | |  | NNT (NNH) | |  | Staff cost / life | |  | Net cost / life | |
| --- | --- | --- | --- | --- | --- | --- | --- | --- | --- | --- | --- | --- | --- | --- | --- | --- | --- |
| Temporary staff availability | Establishment approach | |  | Establishment approach | |  | Establishment approach | |  | Establishment approach | |  | Establishment approach | |  | Establishment approach | |
|  | High (Resilient) | Low (Flexible) |  | High (Resilient) | Low (Flexible) |  | High (Resilient) | Low (Flexible) |  | High (Resilient) | Low (Flexible) |  | High (Resilient) | Low (Flexible) |  | High (Resilient) | Low (Flexible) |
| None | 7.8% | -19.9% |  | -1.4% | 2.4% |  | -4.2% | 13.5% |  | 762 | (219) |  | £40,161 | -£29,842 |  | £20,404 | -£19,976 |
| Limited | 5.7% | -11.0% |  | -1.2% | 1.7% |  | -4.5% | 8.7% |  | 660 | (357) |  | £26,654 | -£28,901 |  | £11,983 | -£17,768 |
| Higher | 4.2% | -5.7% |  | -1.0% | 0.8% |  | -3.8% | 4.7% |  | 828 | (690) |  | £23,359 | -£28,449 |  | £10,488 | -£18,277 |
| Unlimited | 3.2% | -2.2% |  | -0.9% | 0.3% |  | -3.2% | 2.3% |  | 1106 | (2372) |  | £21,375 | -£16,236 |  | £7,670 | -£7,057 |

Table 9: Changes in Costs, effects and cost-effectiveness for resilient and flexible establishments relative to ‘standard’ establishments for varying levels of temporary staff availability (mean and range across three hospital models)

|  | Approach to setting establishment | | | | | | | | |
| --- | --- | --- | --- | --- | --- | --- | --- | --- | --- |
| Temporary staff availability | Resilient (high) | | | |  | Flexible (low) | | | |
| Outcome | Mean | Range | | |  | Mean | Range | | |
| None |  |  |  |  |  |  |  |  |  |
| Staff cost | 7.8% | 10.0% | to | 6.5% |  | -19.9% | -19.6% | to | -20.3% |
| Bed days used | -1.4% | -0.6% | to | -2.5% |  | 2.4% | 2.5% | to | 2.2% |
| Deaths | -4.5% | -4.2% | to | -5.0% |  | 13.4% | 14.5% | to | 12.0% |
| Number needed to treat (harm) | 663 | 764 | to | 512 |  | (222) | (260) | to | (188) |
| Staff cost per life saved | £25,584 | £31,681 | to | £20,987 |  | -£ 23,936 | -£25,384 | to | -£22,721 |
| Net cost per life saved | £ 13,155 | £23,079 | to | £9,699 |  | -£ 16,015 | -£17,473 | to | -£14,887 |
| Limited | |  |  |  |  |  |  |  |  |
| Staff cost | 5.50% | 8.5% | to | 3.8% |  | -10.8% | -9.9% | to | -12.3% |
| Change in bed days used | -1.22% | -0.5% | to | -2.3% |  | 1.7% | 1.8% | to | 1.5% |
| Deaths | -4.5% | -4.1% | to | -4.8% |  | 8.3% | 9.3% | to | 7.1% |
| Number needed to treat (harm) | 665 | 781 | to | 567 |  | (361) | (449) | to | (300) |
| Staff cost per life saved | £ 19,437 | £25,306 | to | £15,870 |  | -£ 21,766 | -£29,290 | to | -£18,719 |
| Net cost per life saved | £ 8,653 | £13,954 | to | £7,277 |  | -£ 21,766 | -£18,377 | to | -£11,343 |
| Higher | | |  |  |  |  |  |  |  |
| Staff cost | 4.0% | 7.0% | to | 2.5% |  | -5.5% | -4.0% | to | -7.8% |
| Change in bed days used | -1.0% | -0.4% | to | -2.0% |  | 0.8% | 1.0% | to | 0.7% |
| Deaths | -3.8% | -2.5% | to | -5.6% |  | 4.4% | 5.6% | to | 3.1% |
| Number needed to treat (harm) | 873 | 1296 | to | 559 |  | (719) | (1,028) | to | (558) |
| Staff cost per life saved | £ 17,230 | £21,745 | to | £14,905 |  | -£ 20,422 | -£31,406 | to | -£17,006 |
| Net cost per life saved | £ 6,451 | £14,249 | to | £4,386 |  | -£ 12,722 | -£19,339 | to | -£9,904 |
| Unlimited | |  |  |  |  |  |  |  |  |
| Staff cost | 2.9% | 6.0% | to | 1.3% |  | -1.6% | 0.1% | to | -4.7% |
| Change in bed days used | -0.9% | -0.2% | to | -1.9% |  | 0.3% | 0.4% | to | 0.2% |
| Deaths | -3.0% | -1.5% | to | -5.4% |  | 1.9% | 3.8% | to | 0.5% |
| Number needed to treat (harm) | 1,272 | 2,065 | to | 580 |  | 2828 | 5921 | to | 829 |
| Staff cost per life saved | £ 15,612 | £19,749 | to | £13,097 |  | -£ 12,722 | -£18,562 | to | £1,882 |
| Net cost per life saved | £ 3,693 | £11,452 | to | £2,022 |  | -£ 4,520 | -£14,130 | to | £6,975 |
